# Phosphate removal combined with acetate supplementation enhances lipid production from water hyacinth by *Cutaneotrichosporon oleaginosum*

**DOI:** 10.1186/s13068-019-1491-y

**Published:** 2019-06-15

**Authors:** Wenting Zhou, Mou Tang, Tao Zou, Na Peng, Mi Zhao, Zhiwei Gong

**Affiliations:** 1 School of Chemistry and Chemical Engineering, Wuhan University of Science and Technology, 947 Heping Road, Wuhan, 430081 People’s Republic of China; 2 HuBei Province Key Laboratory of Coal Conversion and New Carbon Materials, Wuhan University of Science and Technology, Wuhan, 430081 People’s Republic of China; 3 China Carbon Balance Energy and Tech LTD, 1 Jianguomenwai Avenue, Beijing, 100004 People’s Republic of China

**Keywords:** *Cutaneotrichosporon oleaginosum*, Water hyacinth, Acetate, Phosphorus limitation, Microbial lipid

## Abstract

**Background:**

Microbial lipids derived from various lignocellulosic feedstocks have emerged as a promising candidate for the biodiesel industry and a potential substitute for high value-added fats. However, lignocellulosic biomass, especially herbaceous biomass, such as water hyacinth, contains high concentrations of nitrogenous components. These compounds impede microbial lipid production, as lipid biosynthesis is commonly induced by imposing a nutrient deficiency, especially nitrogen starvation. Novel strategies and bioprocesses are pivotal for promoting lipid production from nitrogen-rich biomass.

**Results:**

Here a combined strategy of phosphate removal and acetate supplementation was described for enhanced microbial lipid production on water hyacinth hydrolysates by *Cutaneotrichosporon oleaginosum* (formerly *Cryptococcus curvatus*). Lipid production was significantly improved, when the phosphorus limitation and sugars/acetate co-utilization strategies were used separately. In this case, acetate and glucose were consumed simultaneously. Lipid production was observed by the combination of phosphate removal with acetate supplementation. Lipid titer, content, and yield were determined to be 7.3 g/L, 59.7% and 10.1 g/100 g raw water hyacinth, respectively. These data were increased by 4.2, 4.6, and 4.3 times, respectively, compared to those from the unprocessed hydrolysates. The fatty acid compositions of the resulting lipids bear a marked resemblance to those of rapeseed oil, indicating their applicability to the biodiesel industry.

**Conclusions:**

The combination of phosphate removal and acetate supplementation was successful in significantly enhancing microbial lipid production. This strategy offers a valuable solution for nitrogen-rich lignocellulosic feedstocks utilization, which should foster more economical nitrogen-rich biomass-to-lipid bioprocesses.

## Introduction

Water hyacinth (*Eichhornia crassipes*) is a widespread aquatic weed in sub-tropical and tropical regions. It has been regarded as a serious threat to the biological diversity and ecological equilibrium in recent years because of its extraordinary adaptability and fast growth rate [1, 2]. Water hyacinth contains high amounts of cellulose and hemicellulose, which can liberate fermentable sugars containing glucose, xylose, and other sugars. Moreover, water hyacinth can be relatively easily deconstructed due to its lower degree of lignification than recalcitrant biomass, which includes forestry wastes and agricultural residues [2–4]. Thus, water hyacinth may be explored as a cost-competitive feedstock for fermentation producing biofuels and biochemicals. Recently, water hyacinth has been investigated for bioethanol and biogas production [5–8].

Microbial lipid prepared from various lignocellulosic feedstocks has emerged as a perfect candidate for biodiesel; as well as a potential substitute for high value-added fats [9, 10]. Glucose and xylose, the two principal monosaccharides released from lignocellulosic biomass, have been metabolized for microbial lipid production by various oleaginous species [11–14]. However, lignocellulosic feedstocks naturally contain various amounts of nitrogenous components [15]. Water hyacinth, for example, contains crude proteins up to 13.3% of its dry weight [4]. This high-nitrogen content suggests that the hydrolysates with very low carbon/nitrogen (C/N) molar ratios should not support lipid biosynthesis, as lipogenesis generally occurs under nitrogen deficient conditions [16]. The elimination of nitrogenous components in feedstocks is, therefore, essential for lipid overproduction. Unfortunately, the nitrogenous components within lignocelluloses are technically difficult to remove. Biological means of removing nitrogen has been attempted to promote lipid production. However, nitrogen removal was limited and thus the lipid content remained extremely low [17].

Lipid production is well triggered under phosphorus-limited conditions by *Rhodosporidium toruloides* [18]. When the phosphate content in *Laminaria* residue hydrolysates was removed, the lipid yield and content were increased significantly to 0.16 g/g and 37.6%, respectively [19]. We also note that phosphate content is easily eliminated by precipitation assisted by calcium ions [18]. Thus, phosphorus removal may be explored as a valuable strategy to promote lipid production from nitrogenous substrates. In addition, carbon source supplementation is a very simple approach to increase the C/N ratio and promote lipid accumulation. *Cutaneotrichosporon oleaginosum* (formerly *Cryptococcus curvatus*) has several ideal characteristics for lipid fermentation, especially a wide substrate range and good adaptation to various inhibitors derived from lignocelluloses degradation [20, 21]. Specifically, acetate, a by-product routinely co-generated during anaerobic digestion, syngas fermentation and methane fermentation, has served as a promising carbon source for the lipogenesis of *C. oleaginosum* [22–25]. Moreover, lignocellulosic derived sugars and acetate co-fermentation has been reported to facilitate yeast lipogenesis [11]. Here, the combination of phosphate removal with acetate supplementation was evaluated for lipid production from the enzymatic hydrolysates of water hyacinth pretreated by dilute sulfuric acid. Enhanced lipid production was observed when the phosphorus limitation and the sugars/acetate co-utilization strategies were used separately. Significantly higher lipid production capacities were achieved by the integrated strategy, indicating the cumulative effect of the two strategies. This approach provides a valuable solution for microbial lipid overproduction from nitrogen-rich biomass.

## Materials and methods

### Strain and media

The oleaginous yeast *C. oleaginosum* (formerly *C. curvatus*) ATCC 20509 used in the present work was obtained from the American Type Culture Collection (ATCC). This strain was maintained at 4 °C and propagated twice a month at 30 °C on slants of yeast peptone dextrose (YPD) agar according to a published formula [11]. Yeast inoculums were prepared in the YPD seed medium consisting of 10 g/L yeast extract, 10 g/L peptone, and 20 g/L glucose. The C/N ratio and the C/P ratio were 4.0 and 143.5, respectively.

The nutrients-rich medium included 15.0 g/L glucose, 15.0 g/L xylose, 4.0 g/L (NH_4_)_2_SO_4_, 2.0 g/L yeast extract, 2.7 g/L KH_2_PO_4_, 2.4 g/L Na_2_HPO_4_·12H_2_O, 0.5 g/L MgSO_4_·7H_2_O, 0.1 g/L EDTA, and 1% (v/v) trace element solution. The initial pH was 5.5. The initial C/N ratio was 13.6. The trace element solution was prepared according to a published formula [26].

To study the effects of various phosphate concentrations on the cell growth and lipid production, the nutrients-rich medium was modified as follows: 15.0 g/L glucose, 15.0 g/L xylose, 4.0 g/L (NH_4_)_2_SO_4_, 2.0 g/L peptone, 0.5 g/L MgSO_4_·7H_2_O, 0.1 g/L EDTA, and 1% (v/v) trace element solution. The KH_2_PO_4_ concentration was varied from 0 to 2.0 g/L. K_2_SO_4_ was supplemented to maintain an identical K^+^ concentration according to a published method [18].

For the response surface analysis, the nutrients-rich medium was modified as follows: 15.0 g/L glucose, 15.0 g/L xylose, 4.0 g/L (NH_4_)_2_SO_4_, 0.5 g/L yeast extract, 1.5 g/L peptone, 1.0 g/L Na_2_SO_4_, 1.0 g/L K_2_SO_4_, 0.5 g/L MgSO_4_·7H_2_O, 0.1 g/L EDTA, 1% (v/v) trace element solution; KH_2_PO_4_ concentration was varied from 0 to 0.2 g/L, and acetate concentration was varied from 0 to 15.0 g/L.

All the media were subjected to sterilization by autoclaving for 20 min at 121 °C prior to use.

### Water hyacinth and dilute sulfuric acid pretreatment

Fresh water hyacinth was harvested from the East Lake (Wuhan, China). It was washed to remove the adhering soil, metals, and stones; and then naturally dried using the sunlight. It was then milled and passed through a 40 mesh screen, dried at 105 °C until the weight was constant, and then stored in a desiccator for long-term storage. The chemical composition of the water hyacinth was 21.8% cellulose, 25.3% hemicellulose, 11.6% lignin, and 20.0% crude proteins. Water hyacinth was pretreated using 0.5–2.0% (w/v) dilute sulfuric acid at 120 °C for 60 min. The solid-to-liquid ratio was 1:10 (w/v).

### Enzymatic hydrolysis of dilute acid pretreated water hyacinth

The pretreated slurries were adjusted to pH 4.8 with solid sodium hydroxide or sodium acetate. The enzymatic hydrolysis was carried out at 50 °C, pH 4.8 for 48 h at 8% (w/v) solid loading. Cellulase, β-glucosidase, and xylanase preparations were added at loadings of 15 FPU, 30 CBU and 5 mg per gram water hyacinth, respectively, as described [11].

### Phosphate removal

Phosphate removal was conducted as follows: calcium hydroxide powder was gradually added into the liquid enzymatic hydrolysates until the pH reached pH 10.0. The suspensions were then magnetically stirred for 30 min at ambient temperature and then set for 2 h. Phosphate in the hydrolysates was allowed to react with the added calcium ion. The resulting sediments were eliminated by centrifugation (6000×*g*, 5 min). The liquid hydrolysates were then adjusted to pH 5.5/7.0 before sterilization using sulfuric acid or acetic acid.

### Lipid production on nutrient-rich substrates using various acetate or phosphate concentrations

Yeast precultures were prepared in the YPD seed medium in 250-mL Erlenmeyer flasks for 24 h. Unless otherwise stated, the cultures were shaken at 200 rpm and maintained at 30 °C in an orbital shaking incubator. Precultures (5 mL) were then inoculated into 45 mL of the sterilized media and cultured under identical conditions. The culture media pH was adjusted to the original values (pH 5.5/7.0) at intervals of 12 h.

### Lipid production on various processed water hyacinth hydrolysates

The water hyacinth enzymatic hydrolysates were processed in a boiling water bath for 20 min and separated by centrifugation to remove precipitated proteins and unhydrolyzed residues, generating routine hydrolysates or acetate-rich hydrolysates, respectively. Phosphate was then removed to generate two types of phosphorus-limited hydrolysates, according to the above phosphate removal procedure. These hydrolysates without detoxification and auxiliary nutrients supplementation were adjusted to pH 5.5 (without acetate addition) or pH 7.0 (with acetate addition). They were then sterilized by autoclaving at 121 °C for 20 min. Lipid fermentation was carried out using 45 mL of the sterilized liquid hydrolysates inoculated with 10% (v/v) of the preculture. The fermentations were kept at 30 °C and 200 rpm in a shaking incubator. At the 12-h interval, the pH of the culture media was adjusted to the original value. Unless otherwise specified, all the fermentations were conducted in triplicate. The results were exhibited as the mean values and standard deviation of three independent experiments.

### Analytical method

Acetic acid, glucose, and xylose were measured according to previously published literature [11]. Total reducing sugars (TRS) was determined using the dinitrosalicylate (DNS) method [27]. Total nitrogen was measured according to the Kjeldahl determination with minor modifications as described by Gong and coworkers [20]. The ammonium molybdate spectrophotometric method was used for determination of the phosphorus content [28].

Cellulose and hemicellulose contents of water hyacinth were determined by the detergent extraction method [29]. Lignin content was analyzed according to a standard procedure developed by the National Renewable Energy Laboratory (NREL) [30].

Cell mass, expressed as dry cell weight (DCW), was determined by a gravimetric method [11]. Lipid was extracted twice from the dry cells using a mixture of chloroform and methanol (1:1, v/v) and measured by a gravimetric method [31]. Lipid titer was presented as gram lipid per liter culture broth. Lipid content and lipid yield were defined as gram lipid per gram DCW and gram lipid accumulated per gram substrates (sugars and acetate) consumed, respectively.

To determine the fatty acid compositions, the microbial lipid samples were transesterified with methanol. Then, the resulting fatty acid methyl esters (FAMEs) were measured using a GC-2010Plus gas chromatograph (Shimadzu, Japan) following a standard procedure [11].

## Results and discussion

### Lipid production on the enzymatic hydrolysates of water hyacinth pretreated by dilute sulfuric acid

**Fig. 1:**
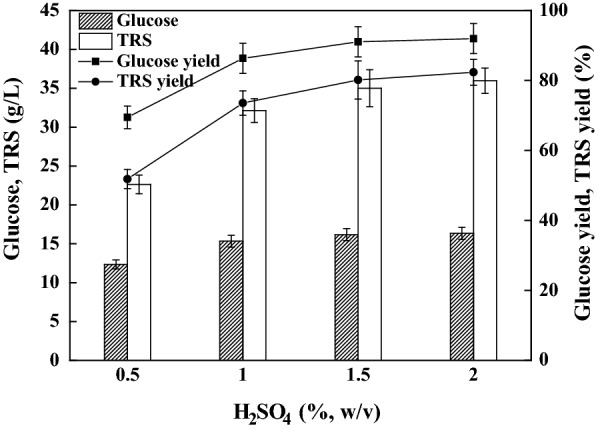
Effects of sulfuric acid loading for dilute acid pretreatment on the enzymatic hydrolysis of the regenerated water hyacinth. The raw water hyacinth was pretreated at 120 °C for 1 h at a solids loading of 10% (w/v). The regenerated samples (8%, w/v) were hydrolyzed at 50 °C for 48 h

Dilute sulfuric acid pretreatment has been extensively described for enhancing the enzyme accessibility of cellulose primarily by deconstructing the hemicellulosic portion of the lignocelluloses into soluble sugars [32]. Sulfuric acid concentration is a crucial factor in this pretreatment. Here, water hyacinth was pretreated with 0.5 to 2.0% (w/v) sulfuric acid solution as described above. The pretreated slurries were then enzymatically hydrolyzed for 48 h at 8% (w/v) solids loading. The results are depicted in Fig. 1. It was clear that higher sulfuric acid concentrations resulted in higher enzymatic hydrolysis yields. Glucose and TRS were as low as 12.3 g/L and 22.6 g/L, respectively, when 0.5% sulfuric acid was used. When the acid loading was doubled, these data achieved 15.3 g/L and 32.1 g/L, respectively. The variances were found to be significant (*P* < 0.05) according to an (ANOVA) analysis of variance and the Tukey’s post hoc test. When sulfuric acid loading increased to 1.5%, these data reached 16.2 g/L and 35.0 g/L, respectively; corresponding to theoretical yields of 83.7% and 80.1%, respectively. The insoluble materials recovered reached 21.6%, indicating small amounts of polysaccharides were not hydrolyzed. When 2% sulfuric acid was loaded, these data slightly increased to 16.3 g/L and 36.0 g/L. However, the enhancement in sugars released was not found to be significant (*P* > 0.05).

**Fig. 2:**
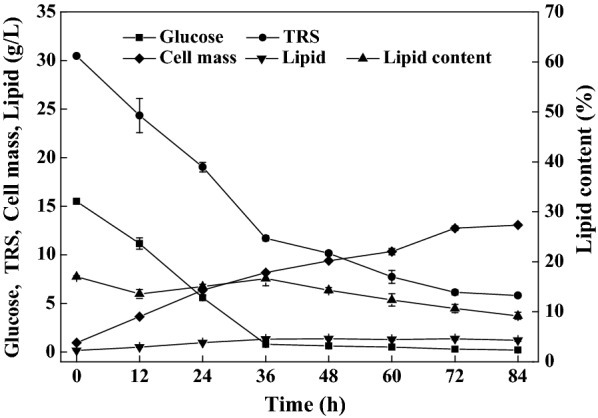
Profiles of substrates consumption, cell growth, and lipid production on the water hyacinth enzymatic hydrolysates by *C. oleaginosum*

Lipid fermentation by *C. oleaginosum* on the enzymatic hydrolysates of water hyacinth pretreated by 1.5% (w/v) sulfuric acid was then conducted. The hydrolysates were directly used for these cultures without detoxification or auxiliary nutrients supplementation. As shown in Fig. 2, sugars (mainly glucose) were consumed quickly within 36 h and the sugars assimilation rate was found to be 0.52 g/L/h. Thereafter, TRS was assimilated at a very low consumption rate, suggesting certain sugars could not be metabolized by *C. oleaginosum*. For example, arabinose is poorly assimilated by *C. oleaginosum*, probably due to arabinose transport deficiency and/or cofactor imbalance of the arabinose oxidoreductase pathway [21]. Indeed, water hyacinth contains 8.9% of arabinan according to a published literature [33]. When the fermentation was stopped at 84 h, the residual TRS was 5.8 g/L, corresponding to 19.0% of the total sugars. Cell mass reached 13.1 g/L, whereas lipid content was only 9.2%. The cell mass yield reached 0.50 g/g, indicating that the hydrolysates contained adequate nutrients to support cell growth. Lipid contents were always lower than 20% during culture, demonstrating that water hyacinth hydrolysates were not proper for lipid biosynthesis. The highest lipid titer was only 1.4 g/L, corresponding to 1.9 g/100 g raw water hyacinth (Table 1, Entry 1).

**Table 1:** Cultivation results of *C. oleaginosum* on various water hyacinth enzymatic hydrolysates

Entry	Phosphate removal	Acetate supplementation	Phosphorus (mg/L)	Nitrogen (g/L)	Initial TRS (g/L)	Cell mass (g/L)	Lipid titer (g/L)	Lipid content (%, w/w)	Lipid yield (g/100 g)	Lipid yield^a^ (g/100 g)
1	×	×	403.4 ± 6.6	2.0 ± 0.1	34.7 ± 0.5	12.7 ± 0.2	1.4 ± 0.1	10.7 ± 0.8	5.6 ± 0.4	1.9 ± 0.2
2	√	×	15.7 ± 0.3	2.0 ± 0.0	33.6 ± 0.4	12.4 ± 0.3	4.5 ± 0.2	35.8 ± 1.7	17.9 ± 0.9	6.2 ± 0.3
3	×	√	389.5 ± 0.7	2.0 ± 0.1	33.0 ± 0.3	11.4 ± 0.4	3.6 ± 0.2	31.4 ± 0.7	9.2 ± 0.5	5.0 ± 0.3
4	√	√	25.8 ± 0.1	2.0 ± 0.0	32.2 ± 0.5	12.2 ± 0.2	7.3 ± 0.1	59.7 ± 0.6	19.6 ± 0.4	10.1 ± 0.2

^a^Lipid yield was calculated as Gram lipid produced per 100 g raw water hyacinth provided

Lipogenesis is thought to be stimulated by nitrogen deficiency [16]. However, the water hyacinth samples contained 3.2% of nitrogen, corresponding to 20% of the crude proteins. The total nitrogen within the hydrolysates reached 2.0 g/L, resulting in a very low C/N ratio (i.e., 0.5), which disfavored lipid overproduction. Furfural, 5-hydroxymethylfurfural, and acetic acid, three compounds known to be toxic, were routinely generated by the dilute acid pretreatment process of lignocellulosic biomass [32]. It should be noted that *C. oleaginosum* showed good adaptation to the water hyacinth hydrolysates, which was consistent with a previous observation by Yu and coworkers [14].

### Lipid production on the enzymatic hydrolysates of water hyacinth under phosphorus limitation

**Fig. 3:**
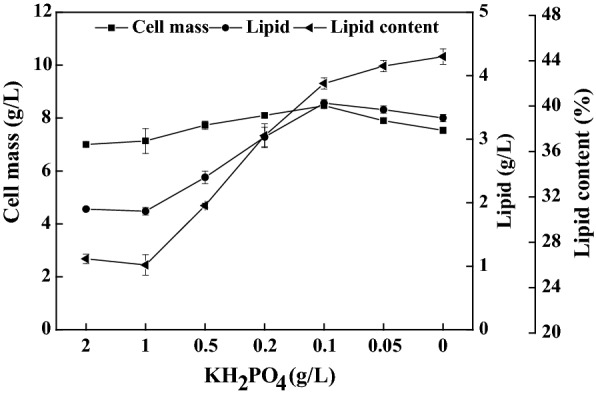
Results of lipid production on the nutrients-rich media by *C. oleaginosum* in the presence of various amounts of phosphate. The initial C/N ratio was 12.3

To determine whether lipid production by *C. oleaginosum* was influenced by the phosphorus limitation, various phosphate levels were investigated and compared. The KH_2_PO_4_ concentrations were reduced from 2 g/L to zero in the media, whereas the C/N ratios maintained as low as 12.3. Lipid titer and content were as low as 1.9 g/L and 26.5%, respectively, when the media contained 2 g/L KH_2_PO_4_ (Fig. 3). Lipid production was not observably perturbed when the KH_2_PO_4_ concentration was decreased to 1 g/L. Interestingly, beneficial effects on lipid overproduction were observed when the KH_2_PO_4_ concentrations ranged from 0.5 g/L to zero (Fig. 3). Lipid titer and content increased by 87.7% and 58.5%, respectively, when the medium contained 0.1 g/L KH_2_PO_4_ and had a C/P ratio of 1360. However, lipid titer slightly decreased from 3.6 to 3.3 g/L when KH_2_PO_4_ was further reduced from 0.1 g/L to zero, whereas lipid content slightly increased from 42.0 to 44.4%.

The TRS and phosphorus concentrations within the water hyacinth hydrolysates were 34.7 g/L and 403.4 mg/L, respectively. The phosphate was then removed by forming precipitation using Ca(OH)_2_ at pH 10.0. As shown in Table 1, TRS and phosphate were decreased by 3.2% and 96.1%, respectively, indicating that the method was efficient for phosphate removal with only minor sugars loss. The total nitrogen concentration was 2.0 g/L, which was identical to the untreated hydrolysates, indicating that the nitrogenous components were 100% conserved in the hydrolysates (Table 1). The following hydrolysates were then used for lipid fermentation. Cell mass was found to be 12.4 g/L when the fermentation was stopped at 72 h. Interestingly, lipid titer, content, and yield achieved 4.5 g/L, 35.8%, and 6.2 g/100 g raw water hyacinth, respectively (Table 1, Entry 2). These data were increased by 2.2, 2.3, and 2.3 times, respectively, compared to those obtained from the untreated hydrolysates.

Phosphorus limitation has been recommended to mediate lipid overproduction on nitrogen-rich substrates [18, 19]. The mechanism of lipid overproduction under phosphate limitation has been revealed by the multi-omics results of studies of *R. toruloides* fermentation [34]. The phosphate relevant metabolism, ribonucleic acid (RNA) degradation and triacylglycerols (TAG) biosynthesis are activated, whereas the tricarboxylic acid (TCA) cycle and ribosome biosynthesis are inhibited under phosphate limitation, which channels carbon flux to lipid biosynthesis. Here, superior lipid production by *C. oleaginosum* was observed on the hydrolysates treated with phosphate elimination. Thus, phosphate removal may be explored as a simple and cost-effective strategy to advance lipid production from lignocellulosic feedstocks with abundant nutrients, especially nitrogen.

### Lipid production on the water hyacinth enzymatic hydrolysates with acetate supplementation

**Fig. 4:**
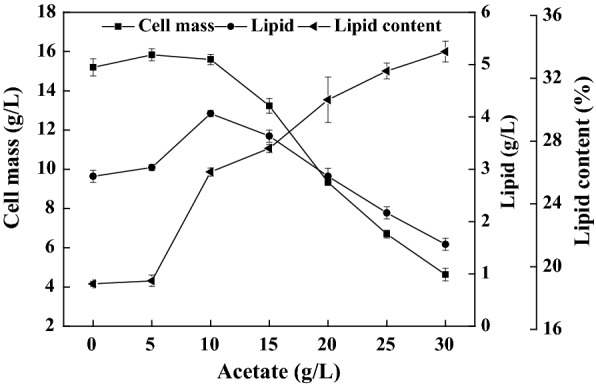
Results of lipid production on the nutrients-rich media by *C. oleaginosum* in the presence of various amounts of acetate. The initial C/N ratio was set at 13.6

To demonstrate the effectiveness of carbon source supplementation on lipid accumulation, *C. oleaginosum* was cultured on the nutrients-rich media with acetate supplementation. The results are shown in Fig. 4. When *C. oleaginosum* was cultured on the nitrogen-rich medium containing a C/N ratio of 13.6, cell mass reached as high as 15.2 g/L. However, lipid titer and content were reduced to 2.9 g/L and 18.9%, respectively, indicating these carbon sources were mainly channeled into cell proliferation. It was clear that positive effects on the lipogenesis were observed when acetate was varied from 5 to 20 g/L (Fig. 4). When 10 g/L acetate was added, lipid titer and content increased significantly (*P* < 0.05) to 4.1 g/L and 26.0%, respectively. However, cell mass and lipid production were both gradually decreased when the additional acetate was increased from 10 to 30 g/L, albeit the lipid content further increased from 26.0 to 33.7%. Although lipid biosynthesis was accelerated, lipid production was decreased because cell proliferation was severely reduced when acetate concentrations exceeded 25 g/L. Xylose was nearly not consumed when the acetate supplementation was as high as 25 g/L, suggesting *C. oleaginosum* utilized glucose and xylose sequentially. Indeed, glucose repression existed when these two sugars were co-utilized for lipid fermentation [35]. The presence of acetate did not alter the sequential utilization of the mixture of glucose and xylose [11].

Sodium acetate was added to the slurry of the pretreated water hyacinth to neutralize the sulfuric acid until the final pH was adjusted to pH 4.8. Then, the whole slurry was enzymatically hydrolyzed to generate hydrolysates rich in sugars and acetate. The initial pH of the liquid hydrolysates was adjusted to pH 7.0. TRS and acetate concentrations of 29.0 g/L and 15.5 g/L, respectively, were found in the water hyacinth hydrolysates. Interestingly, lipid titer, content, and yield reached 3.6 g/L, 31.4%, and 5.0 g/100 g raw water hyacinth, respectively, at the end of the culture (Table 1, Entry 3), which were all significantly improved with respect to those obtained from the hydrolysates without acetate supplementation. It was concluded that co-utilization of water hyacinth hydrolysates and acetate was an effective method to promote lipid production.

Recently, acetic acid has been reported to be co-assimilated with xylose. It is suggested that acetic acid exerts beneficial effects on xylose fermentation by assisting the redox balance [36]. Our previous work demonstrated that acetate and sugars were simultaneously assimilated by *C. oleaginosum* to produce lipids [11]. Thus, suitable acetate supplementation is a good choice to advance lipid production on nutrients-rich substrates.

### Combination of phosphate removal with acetate supplementation for lipid production from water hyacinth enzymatic hydrolysates

Lipid production was significantly improved, when the phosphorus limitation and the sugars/acetate co-utilization strategies were used separately. However, the enhancement remained far from satisfactory. Here, a central composite face-centered design and response surface methodology was applied to optimize the two variables for lipid production [37, 38]. A set of 13 experiments were performed and the results are presented in Table 2. The results were then subjected to ANOVA and residual analysis to check the adequacy of the constructed quadratic models for lipid titer and lipid content, respectively (Tables 3 and 4). The models were significant (*P* < 0.0001), indicating their suitability for explaining the system behavior. Lack of fit, an important parameter to check the model, was found as insignificant (*P* > 0.05) implying the fitness of the models. As shown in Tables 3 and 4, the interaction between *A* and *B* had significant (*P* < 0.05) influences on both lipid titer and content. In the models for lipid titer and lipid content, *R*^2^ and Adj *R*^2^ were always higher than 0.99, indicating that the sample variation over 99% for the lipid production was attributed to the independent variables. *A*, *A*^2^ and *B*^2^ were significant (*P* < 0.05) for lipid titer (Table 3). *A* and *B* were significant with a probability higher than 99.99% (*P* < 0.0001) for lipid content (Table 4). The predicted maximum lipid titer and content were 6.3 g/L (with 0 g/L phosphate and 6.9 g/L acetate) and 57.5% (with 0 g/L phosphate and 15 g/L acetate), respectively.

**Table 2:** The experimental design and results of the central composite design

Run	Factors		Responses	
	*A* (KH_2_PO_4_, g/L)	*B* (HAc, g/L)	*Y*1 (lipid titer, g/L)	*Y*2 (lipid content, %)
1	0 (− 1)	0 (− 1)	5.4	48.3
2	0.2 (+ 1)	0 (− 1)	3.1	28.8
3	0 (− 1)	15.0 (+ 1)	5.1	57.5
4	0.2 (+ 1)	15.0 (+ 1)	3.5	33.2
5	0 (− 1)	7.5 (0)	6.3	54.3
6	0.2 (+ 1)	7.5 (0)	4.3	32.5
7	0.1 (0)	0 (− 1)	3.8	34.8
8	0.1 (0)	15.0 (+ 1)	3.8	40.0
9	0.1 (0)	7.5 (0)	4.9	37.7
10	0.1 (0)	7.5 (0)	4.7	36.8
11	0.1 (0)	7.5 (0)	4.7	36.7
12	0.1 (0)	7.5 (0)	4.8	37.4
13	0.1 (0)	7.5 (0)	4.7	36.8

**Table 3:** Analysis of variances for lipid titer

Source	Sum of squares	*df*	Mean square	*F* value	*P* value Prob > *F*
Model	8.74	5	1.75	393.96	< 0.0001
*A*-KH_2_PO_4_	5.80	1	5.80	1307.39	< 0.0001
*B*-HAc	0.0017	1	0.0017	0.38	0.5594
AB	0.12	1	0.12	27.60	0.0012
*A*^2^	0.69	1	0.69	154.53	< 0.0001
*B*^2^	2.77	1	2.77	624.53	< 0.0001
Residual	0.031	7	0.0044		
Lack of fit	0.0031	3	0.0010	0.15	0.9272
Pure error	0.028	4	0.007		
Corrected total	8.77	12			
*R* ^2^	0.9965				
Adjusted *R*^2^	0.9939				
Predicted *R*^2^	0.9923				
Adequate precision	69.8259

**Table 4:** Analysis of variances for lipid content

Source	Sum of squares	*df*	Mean square	*F* value	*P* value Prob > *F*
Model	868.12	5	173.62	348.02	< 0.0001
*A*-KH_2_PO_4_	717.23	1	717.23	1437.66	< 0.0001
*B*-HAc	58.91	1	58.91	118.08	< 0.0001
AB	5.76	1	5.76	11.55	0.0115
*A*^2^	79.72	1	79.72	159.79	< 0.0001
*B*^2^	1.09	1	1.09	2.18	0.1833
Residual	3.49	7	0.50		
Lack of fit	2.60	3	0.87	3.89	0.1114
Pure error	0.89	4	0.22		
Corrected total	871.61	12			
*R* ^2^	0.9960				
Adjusted *R*^2^	0.9931				
Predicted *R*^2^	0.9749				
Adequate precision	58.6297				

**Fig. 5:**
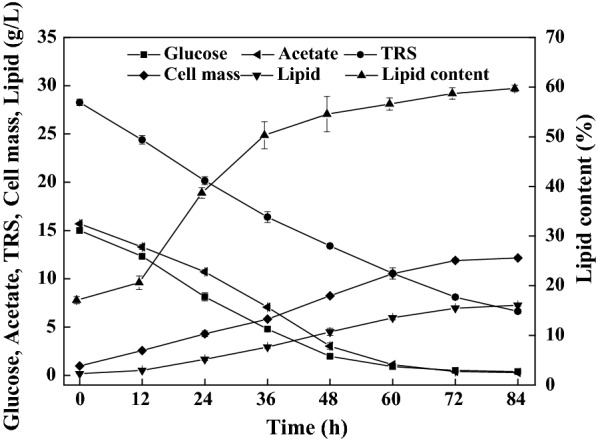
Profiles of substrates consumption, cell growth and lipid production by *C. oleaginosum*. Cells were cultivated on the water hyacinth enzymatic hydrolysates with phosphate removal and acetate supplementation

**Fig. 6:**
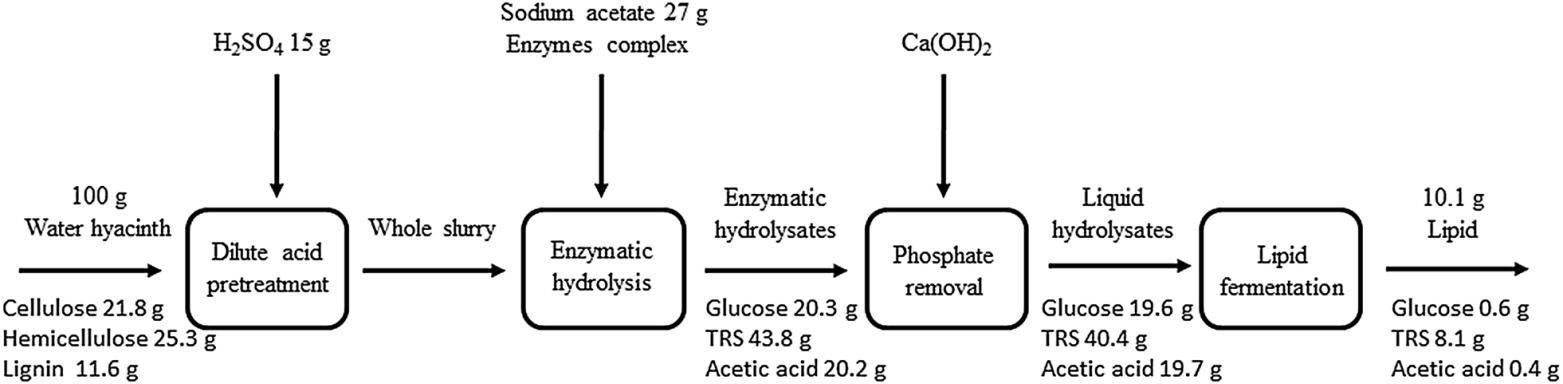
Water hyacinth to microbial lipid mass balance analysis. The analysis was based on dilute sulfuric acid as feedstock pretreatment technology and *C. oleaginosum* as the lipogenic strain. Enzymatic hydrolysis was conducted at a solids loading of 8% (w/v)

The combination of phosphate removal with acetate supplementation was further investigated for lipid production and the results are presented in Fig. 5. Simultaneous assimilation of glucose and acetate prevalent in water hyacinth hydrolysates were observed (Fig. 5). Lipid accumulation increased constantly over time. When the fermentation was stopped at 84 h, lipid titer and content were up to 7.3 g/L and 59.7%, respectively (Table 1, Entry 4). These values were 5.2 and 5.6 times those found from the untreated hydrolysates. The lipid yield was up to 19.9 g/100 g carbon source consumed, indicating that the lipid biosynthesis was triggered. Acetate probably contributed a lot to the lipid biosynthesis, since it was almost fully consumed (Fig. 5). We note that acetate has served as a promising substrate for lipid overproduction by *C. oleaginosum* [22–24]. Lipid titer, content, and yield were significantly (*P* < 0.05) improved compared to those achieved by the separately phosphate removal or acetate supplementation strategy. 10.1 g microbial lipid could be generated from 100 g raw water hyacinth according to the mass balance analysis (Fig. 6), which was 5.3 times that obtained from the untreated hydrolysates. Thus, the combined strategy achieved cumulative effects and rendered higher lipid production.

Results using various lignocellulosic hydrolysates for microbial lipid fermentation by various oleaginous species are summarized in Table 5. Compared to rice straw hydrolysates [39], sugarcane bagasse hydrolysates [40], wheat straw hydrolysates [14, 41], corn stover hydrolysates [17, 20, 42, 43], corncob residues hydrolysates [44], waste paper hydrolysates [45], laminaria residues hydrolysates [19], groundnut shell hydrolysates [46], cardoon stalks hydrolysates [47], and elephant grass hydrolysates [48], the present water hyacinth hydrolysates demonstrated inferior results of lipid production. The herbaceous biomass was nutrients-rich and resulted in very low lipid production. Surprisingly, the combination of phosphate removal with acetate supplementation resulted in high lipid content and productivity of 59.7% and 0.087 g/L/h, respectively, suggesting this combined strategy was very powerful for microbial lipid overproduction on the nitrogen-rich materials (Table 2). However, the lipid titer and productivity remained significantly lower than those obtained by oleaginous yeasts grown on lignocellulosic hydrolysates using the two-stage culture mode or the fed-batch culture mode [41, 43].

**Table 5:** Lipid production from various lignocellulosic biomass by different oleaginous species

Oleaginous yeasts	Feedstocks	Cell mass (g/L)	Lipid titer (g/L)	Lipid content (%, w/w)	Lipid productivity (g/L/h)	References
*Geotrichum fermentans* ^a^	Rice straw hydrolysates	28.6	11.5	40.1	0.059	[39]
*Yarrowia lipolytica*	Sugarcane bagasse hydrolysates	11.4	6.7	58.5	0.073	[40]
*Rhodotorula toruloides* ^b^	Laminaria residue hydrolysates	12.7	4.8	37.6	0.067	[19]
*R. toruloides*	Wheat straw hydrolysates	64.5	39.5	61.3	0.334	[41]
*R. toruloides*	Corn stover hydrolysates	15.2	5.5	36.4	0.035	[42]
*Lipomyces tetrasporus*	Corn stover hydrolysates	54.3	29.0	53.4	0.215	[43]
*Cutaneotrichosporon cutaneum* ^c^	Corn stover hydrolysates	15.4	3.1	23.5	0.052	[17]
*C. cutaneum*	Corncob residues hydrolysates	38.4	12.3	38.4	0.064	[44]
*C. cutaneum*	Elephant grass hydrolysates	22.8	5.5	24.0	0.038	[48]
*Vishniacozyma psychrotolerans* ^d^	Groundnut shell hydrolysates	13.7	6.3	46.0	0.044	[46]
*Solicoccozyma terricola*	Cardoon stalks hydrolysates	23.8	13.2	55.6	0.071	[47]
*C. oleaginosum*	Wheat straw hydrolysates	17.2	5.8	33.5	0.040	[14]
*C. oleaginosum*	Corn stover hydrolysates	11.8	4.6	39.4	0.080	[20]
*C. oleaginosum*	Waste paper hydrolysates	15.2	5.8	37.8	0.080	[45]
*C. oleaginosum*	Water hyacinth hydrolysates	12.7	1.4	10.7	0.019	This study
*C. oleaginosum* ^e^	Water hyacinth hydrolysates	12.2	7.3	59.7	0.087	This study

^a^Formerly *Trichosporon fermentans*

^b^Formerly *Rhodosporidium toruloides*

^c^Formerly *Trichosporon cutaneum*

^d^Formerly *Cryptococcus psychrotolerans*

^e^Water hyacinth hydrolysates were processed with phosphate removal and acetate supplementation

Microbial lipid produced by *C. oleaginosum* using the integrated strategy was transesterified with methanol and the resulting FAMEs were analyzed by GC. The fatty acid species found consisted of 1.4% myristic acid, 48.4% palmitic acid, 1.0% palmitoleic acid, 3.0% stearic acid, 43.0% oleic acid, and 2.5% linoleic acid. Specifically, palmitic acid and oleic acid were the predominant components. The lipid samples had a similar fatty acid composition to those of rapeseed oil, demonstrating that the microbial lipid should be a perfect candidate for sustainable production of biodiesel [49].

## Conclusions

The enzymatic hydrolysates of water hyacinth not receiving detoxification and auxiliary nutrients supplementation was found suitable for cell growth, but not lipid accumulation of *C. oleaginosum*. The combination of phosphate removal with acetate supplementation was effective for significantly promoting lipid titer, content, and yield. Acetate and glucose were consumed simultaneously. The combined strategy offers a promising solution for utilization of nitrogen-rich lignocellulosic feedstocks, which should enable development of more effective nitrogen-rich biomass-to-lipid bioprocesses.

## Data Availability

All appropriate data for this study has been included in the manuscript.

## Abbreviations

C/N: carbon-to-nitrogen
FPU: filter paper unit
CBU: cellobiose unit
ATCC: the American Type Culture Collection
YPD: yeast peptone dextrose
TRS: total reducing sugar
DCW: dry cell weight
DNS: dinitrosalicylate
NREL: the National Renewable Energy Laboratory
FAMEs: fatty acid methyl esters
ANOVA: an analysis of variance
RNA: ribonucleic acid
TAG: triacylglycerols
TCA: tricarboxylic acid
